# Genetic Diversity of *Salmonella enterica* subsp. *enterica* Serovar Enteritidis from Human and Non-Human Sources in Portugal

**DOI:** 10.3390/pathogens13020112

**Published:** 2024-01-26

**Authors:** Célia Leão, Leonor Silveira, Ana Usié, Joana Gião, Lurdes Clemente, Patricia Themudo, Ana Amaro, Angela Pista

**Affiliations:** 1 Laboratory of Bacteriology and Mycology, Department of Antimicrobial Resistance, National Institute of Agrarian and Veterinary Research (INIAV, IP), 2780-157 Oeiras, Portugal; celia.leao@iniav.pt (C.L.); joana.silva@iniav.pt (J.G.); lurdes.clemente@iniav.pt (L.C.); patricia.themudo@iniav.pt (P.T.); ana.amaro@iniav.pt (A.A.); 2 MED—Mediterranean Institute for Agriculture, Environment and Development, 7006-554 Évora, Portugal; 3 National Reference Laboratory for Gastrointestinal Infections, Department of Infectious Diseases, National Institute of Health Doutor Ricardo Jorge, 1649-016 Lisbon, Portugal; leonor.silveira@insa.min-saude.pt; 4 Department of Animal Genomics and Bioinformatics, Centro de Biotecnologia Agrícola e Agro-Alimentar do Alentejo (CEBAL), Instituto Politécnico de Beja (IPBeja), 7801-908 Beja, Portugal; ana.usie@cebal.pt; 5 MED—Instituto Mediterrâneo para a Agricultura, Ambiente e Desenvolvimento & CHANGE–Global Change and Sustainability Institute, CEBAL, 7801-908 Beja, Portugal; 6 CIISA—Centre for Interdisciplinary Research in Animal Health, Faculty of Veterinary Science, 1300-477 Lisbon, Portugal

**Keywords:** salmonellosis, foodborne, pathogenicity, one health, WGS

## Abstract

*Salmonella enterica* subsp. *enterica* serovar Enteritidis (*S.* Enteritidis) is one of the leading causes of foodborne infections associated with broilers and laying hens. Portugal has had the lowest notification rates of salmonellosis in recent years, due to the vaccinations of layer and breeder flocks and strict compliance with biosecurity measures. However, data about the genetic diversity of *S.* Enteritidis in Portugal are scarce. In this study, 102 *S.* Enteritidis isolates selected from human (*n* = 63) and non-human sources (*n* = 39) were characterized by serotyping, antimicrobial susceptibility, and whole genome sequencing. The *S.* Enteritidis population was mainly resistant to fluoroquinolones, and a sole isolate showed resistance to extended-spectrum cephalosporins. ST11 was the most frequent sequence type, and three novel STs from human isolates (ST9236, ST4457, and ST9995) were assigned. Several *Salmonella* pathogenic islands (SPI) and Putative SPI were present in the genomes, namely SPI-1, 2, 3, 4, 5, 9, 10, 12, 13, and 14, C63PI, CS54_island, and 170 virulence genes were identified. The phylogenetic analysis revealed that strains from Portugal are genetically heterogeneous regarding sample type, collection date, and genetic content. This study increases the available data, essential to a better characterization of strains in a global context.

## 1. Introduction

Salmonellosis remains the second most frequent foodborne infection in the European Union (EU), with 60,494 confirmed cases in 2021 [1]. Although more than 2600 serovars have been described to date, the three most commonly reported in 2021 were Enteritidis, Typhimurium, and monophasic Typhimurium (1,4,[5],12:i:-), accounting for more than 70% of the human cases acquired in the EU [2]. 

*Salmonella enterica* subsp. *enterica* serovar Enteritidis (*S.* Enteritidis) infection is associated with different sources, mainly broilers and laying hens [2], with eggs and egg products being the primary sources of outbreaks in 2021 [3]. At the EU level, the importance of poultry products in the epidemiology of human infection is underlined by the recurrent high number of Member States that, under their national control plans, did not meet the established reduction target of 2% for the top five *Salmonella* serovars (Enteritidis, Typhimurium, Virchow, Infantis, and Hadar) in laying hens and breeding flocks and 1% for the other poultry populations [2]. Broiler meat is another source of infection, although the contamination of broiler farms has been increasingly associated with other serovars, like Infantis [2,4]. In Portugal, the prevalence of *S.* Enteritidis observed in the different poultry production sectors was 1.1% for layers, 0.02% for broilers, and null in breeding stock and fattening turkeys [2], with prevalence values far below the established limits. The vaccination of all layer and breeder flocks, as well as the compliance with strict biosecurity measures, sharply decreased the prevalence of infected flocks, enabling Portugal to be among the EU countries reporting the lowest notification rates of salmonellosis in the last few years (≤4.6 cases per 100,000 population) [2]. 

Nevertheless, *Salmonella* is the most common foodborne agent in the EU associated with hospitalizations, with more than 10,000 cases in 2021 [2], still representing a high disease burden. Surveillance systems are essential in reducing the clinical and economic burdens of foodborne diseases [5]. Although traditional serotyping is still being used, in recent years, reference laboratories have been adopting a multi-locus sequence-typing (MLST) approach to complement traditional typing [5,6,7]. Recently, whole genome sequencing (WGS) has become of paramount importance in the surveillance of infectious pathogens of public health importance, due to its high throughput and discriminatory power that allow not only the characterization of isolates according to serovar, pathotype, plasmid content, and antimicrobial and virulence profiles, but also enable cluster detection, source tracking, and outbreak investigation [5]. 

In Portugal, data about the genetic diversity of *S.* Enteritidis are scarce. Furthermore, studies from the One Health perspective are yet to be performed. Thus, the main goal of this study was to perform a genomic characterization of this serovar using whole genome sequencing (WGS) technology to analyze the genomic diversity and relationship of *Salmonella* Enteritidis circulating strains isolated from human and non-human sources. 

## 2. Materials and Methods

### 2.1. Bacterial Isolates

A total of 102 *S.* Enteritidis isolates selected from human (*n* = 63) and non-human sources (*n* = 39), obtained between 2012 and 2020, were analyzed at the National Institute of Health Doutor Ricardo Jorge (INSA) and the National Institute of Agrarian and Veterinary Research (INIAV), respectively, concerning serotyping and genetic characterization as part of the national surveillance systems for *Salmonella*. The collection of clinical human isolates and non-human routine monitoring program isolates included five isolates per year from 2012 to 2020, prioritizing those with an antimicrobial resistance phenotype, particularly to beta-lactams, fluoroquinolones, and polymyxins (according to the rationale established in the ADONIS OHEJP). Therefore, this sampling strategy was not representative of the total number of *Salmonella* Enteritidis cases in Portugal. Isolates of human origin were recovered from fecal (*n* = 58) and blood (*n* = 5) samples, while the isolates of non-human origin were of poultry origin recovered from the environment (boot swabs), collected at broiler (*n* = 8) and layer (*n* = 10) poultry farms; from feed collected at feeding mills (*n* = 3); and from food samples (meat, carcass swabs, and neck skin) collected at processing plants, retail stores, and supermarkets (*n* = 18). 

### 2.2. Serotyping of Salmonella Isolates

*Salmonella* isolates were serotyped using the slide agglutination method, based on the detection of their somatic, flagellar, and capsular antigens, according to the Kauffmann–White–Le Minor scheme [8] (SSI Diagnostica, Hilleröd, Denmark; Sifin, Berlin, Deutschland). The Sven Gard method was used for phase inversion. Whenever needed, subspecies differentiation was confirmed by the biochemical test with D-tartrate, malonate, and dulcitol broths. 

### 2.3. Antimicrobial Susceptibility Testing

Fifty-three isolates of human origin (ten isolates were irrecoverable) were tested by disc diffusion, according to the Kirby–Bauer method and following the European Committee on Antimicrobial Susceptibility Testing (EUCAST) recommendations [9], for the following antimicrobials: ampicillin (AMP), amoxicillin-clavulanic acid (AMC), azithromycin (AZT), cefepime (FEP), cefotaxime (CTX), cefoxitin (FOX), ceftazidime (CZD), ceftriaxone (CRO), chloramphenicol (CHL), gentamicin (GEN), meropenem (MEM), nalidixic acid (NAL), pefloxacin (PEF), sulfamethoxazole (SMX), tetracycline (TET), tigecycline (TGC), and trimethoprim (TMP). Additionally, susceptibility to colistin (COL) was tested using the microdilution technique, performed according to CLSI recommendations [10]. Results were interpreted according to the EUCAST clinical breakpoints and epidemiological cut-offs (ECOFFs) whenever clinical breakpoints were unavailable [11].

All isolates of non-human origin were tested for the determination of minimum inhibitory concentrations (MIC) using the microdilution method and commercial standardized microplates (EUVSEC3, SensititreTM, Thermofisher ScientificTM, Loughborough, UK). Additionally, the EUVSEC2 microplate (SensititreTM, Thermofisher ScientificTM, Loughborough, UK) was used to test presumptive beta-lactam-resistant isolates. Results were interpreted according to the EUCAST epidemiological cut-off values, except for colistin, which is not determined, and therefore, MIC > 2 mg/L was used [12]. *Escherichia coli* ATCC 25922 was used as the quality control strain. 

### 2.4. Whole Genome Sequencing and Bioinformatics Analysis

**Figure 1:**
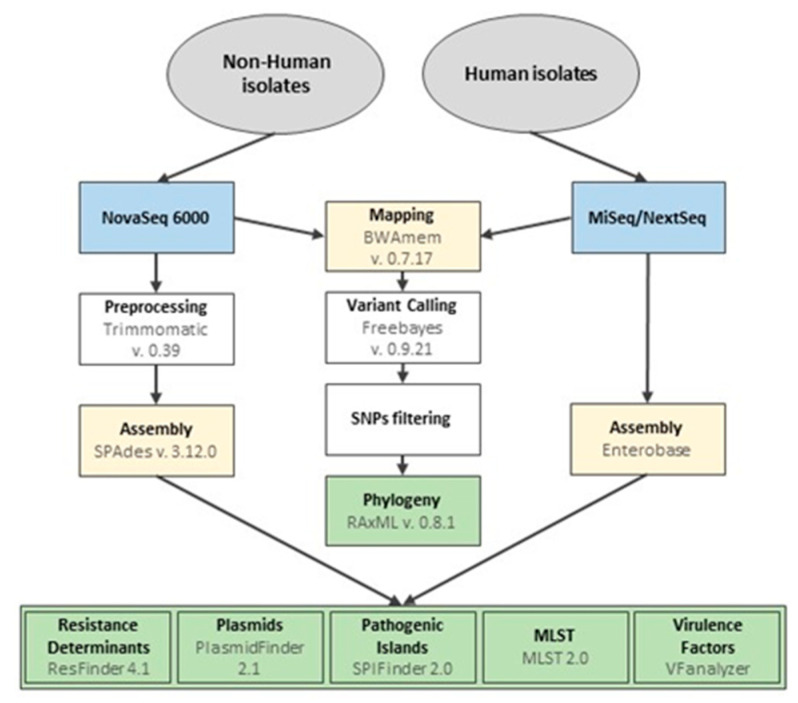
Workflow of the Illumina DNA sequence data analysis. The pipeline included the Illumina platform used for sequencing isolates from human and non-human sources and the strategy used for the assembly of reads for the subsequent analysis using CGE tools and for mapping and phylogeny analysis based on SNPs.

Genomic DNA extraction from fresh cultures of human clinical isolates was achieved using the ISOLATE II Genomic DNA Kit (Bioline, London, UK) and quantified in the Qubit fluorometer (Invitrogen, Waltham, MA, USA) with the dsDNA HS Assay Kit (Thermo Fisher Scientific, Waltham, MA, USA), according to the manufacturer’s instructions. Libraries were prepared with the NexteraXT library preparation protocol (Illumina, San Diego, CA, USA), followed by cluster generation and paired-end sequencing (2 × 250 bp or 2 × 150 bp) on either a MiSeq or a NextSeq 550 instrument (Illumina), according to the manufacturer’s instructions. The workflow of the Illumina DNA sequence data analysis is summarized in Figure 1. Sequencing reads were submitted to the Qassembly pipeline (v 3.61) of EnteroBase v1.1.3 [7,13,14] to generate high-quality assemblies and were deposited in the European Nucleotide Archive (ENA) under the bioproject PRJEB32515. Genomic DNA extraction from isolates of non-human origin was carried out using the PureLink^®^ Genomic DNA kit, following the gram-negative bacterial cell lysate protocol (Invitrogen, Carlsbad, CA, USA), with minor modifications to the manufacturers’ instructions. Briefly, the incubation period at 55 °C was performed for 90 min, and the DNA was eluted with 50 µL of Tris-HCl buffer pH 8.5. DNA quality and quantity were assessed using a spectrophotometer (Nanodrop^®^ 2000, Thermo Scientific, Waltham, MA, USA) and QuantusTM fluorometer using the QuantiFluor^®^ ONE dsDNA Dye kit (Promega, Madison, WI, USA), according to manufacturers’ recommendations. Library preparation and DNA sequencing were performed by Novogene Europe, Cambridge, UK, using Illumina HiSeq sequencing technology (NovaSeq 6000 S2 PE150 XP sequencing mode) to produce paired-end reads with a length of 150 bp. Raw reads were deposited at ENA under project accession number PRJEB44445. Sequencing raw data quality was assessed, and reads were pre-processed and assembled according to Leão et al., 2022 [15]. 

Bioinformatics analysis was performed using tools available at the Center for Genomic Epidemiology (CGE) (https://cge.cbs.dtu.dk; https://bitbucket.org/genomicepidemiology/workspace/projects/CGE accessed on 12 September 2023). For the identification of acquired antimicrobial resistance genes, ResFinder 4.1 was used; chromosomal point mutations were identified with PointFinder [16,17,18,19]; plasmid replicons were identified with PlasmidFinder 2.1 [18,19,20]; and multi-locus sequence types were identified with MLST 2.0 [18,19,21], with default settings. Novel sequence types (STs) were attributed using EnteroBase. Additionally, the serotype and *Salmonella* pathogenicity islands (SPIs) were also determined using SeqSero v1.2 [22] and SPIFinder v2.0 [23], respectively, with default settings. The Comprehensive Antibiotic Resistance Database (CARD) [24] was used to complement the characterization of the isolates’ genomic content. Virulence factors were characterized using the VFanalyzer tool (http://www.mgc.ac.cn/cgi-bin/VFs/genus.cgi?Genus=Salmonella accessed on 12 September 2023) [25] from the Virulence Factors of Pathogenic Bacteria (VFDB) server, and a heatmap visualization of the presence/absence matrix of virulent factors was generated using the R package (R v 4.3.0) ComplexHeatmap [26]. 

A phylogenetic analysis based on single-nucleotide polymorphisms (SNPs) present in the genomes was conducted with isolates from this study using *S.* Enteritidis NCTC13349 (accession number GCF_900456795.1) as the reference genome. Simultaneously, a second approach was conducted with our isolates and 197 other isolates from multiple origins described by Cao et al., 2023 [27]. Reads were mapped against the reference genome using BWA v. 0.7.17-r1188, based on the BWA-MEM algorithm, with default parameters [28]. Only reads with a mapping quality of 25 or above were kept for variant calling, performed with Freebayes v. 0.9.21 with default parameters [29]. The raw variants identified were then filtered by keeping only SNP variants with a minimum quality of 30 and minimum depth coverage per sample of 10. Additionally, SNPs called within a 10-base vicinity of each other were excluded. Maximum likelihood trees were created using RaXML v. 0.8.1 [30]. The graphical representation and tree annotation were performed using iTOL, Interactive Tree of Life v6 [31].

## 3. Results

### 3.1. Characterization of Antimicrobial Resistance

**Figure 2:**
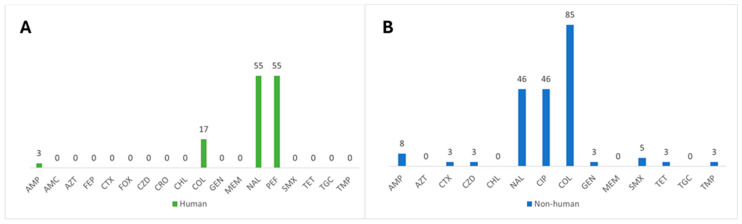
Percentages of antimicrobial susceptibility of *S.* Enteritidis from human (**A**) and non-human (**B**) origins. AMP, ampicillin; AMC, amoxicillin-clavulanic acid; AZT, azithromycin; FEP, cefepime; CTX, cefotaxime; FOX, cefoxitin; CZD, ceftazidime; CRO, ceftriaxone; CHL, chloramphenicol; GEN, gentamicin; MEM, meropenem; NAL, nalidixic acid; PEF, pefloxacin; SMX, sulfamethoxazole; TET, tetracycline; TGC, tigecycline; and TMP, trimethoprim.

In this analysis, 102 *S.* Enteritidis isolates were studied, 63 from human origins and 39 from non-human origins. The antimicrobial resistance frequencies of *S.* Enteritidis are summarized in Figure 2. Isolates of human origin showed 54.7% (29/53) resistance to fluoroquinolones, 17.0% (9/53) resistance to colistin, and 2.8% (2/53) resistance to ampicillin. Resistances to amoxicillin-clavulanic acid, macrolides, cephalosporins, carbapenems, phenicol, aminoglycosides, folate pathway inhibitors, tetracycline, and glycylcycline were not detected, nor were cases of multidrug resistance. Overall, 41.5% (22/53) of the isolates were fully susceptible to the antimicrobials tested. Regarding isolates from non-human sources, high frequencies of resistance to colistin (84.6%, 33/39) and fluoroquinolones (46.2%, 18/39) were verified, and only one bacterial isolate displayed resistance to third-generation cephalosporins. Multidrug resistance was low (7.7%, 3/39). 

### 3.2. Genome Analysis

MiSeq and NextSeq sequencing platforms generated 4,656,832 to 4,802,930 reads with a length of about 250 bp. The assemblies originated between 30 and 254 contigs, longer than 500 bp, an N50 of about 276 Mbp, and a genome size of about 4.6 Gb, with about 52% guanidine–cytosine (GC) content. The NovaSeq 6000 sequencing platform generated 4,703,307 to 8,315,355 reads, with a length of about 150 bp. After the pre-processing, 97.7% to 99.2% of reads remained in the dataset to further bioinformatics steps. The number of contigs over 500 bp produced by the assembly was between 20 and 28, with an N50 of 490 Mbp, genome size of 4.7 Gb, and 52% GC content. 

In silico serotyping confirmed that all isolates were *S. enterica* subsp. *enterica* serovar Enteritidis. 

**Figure 3:**
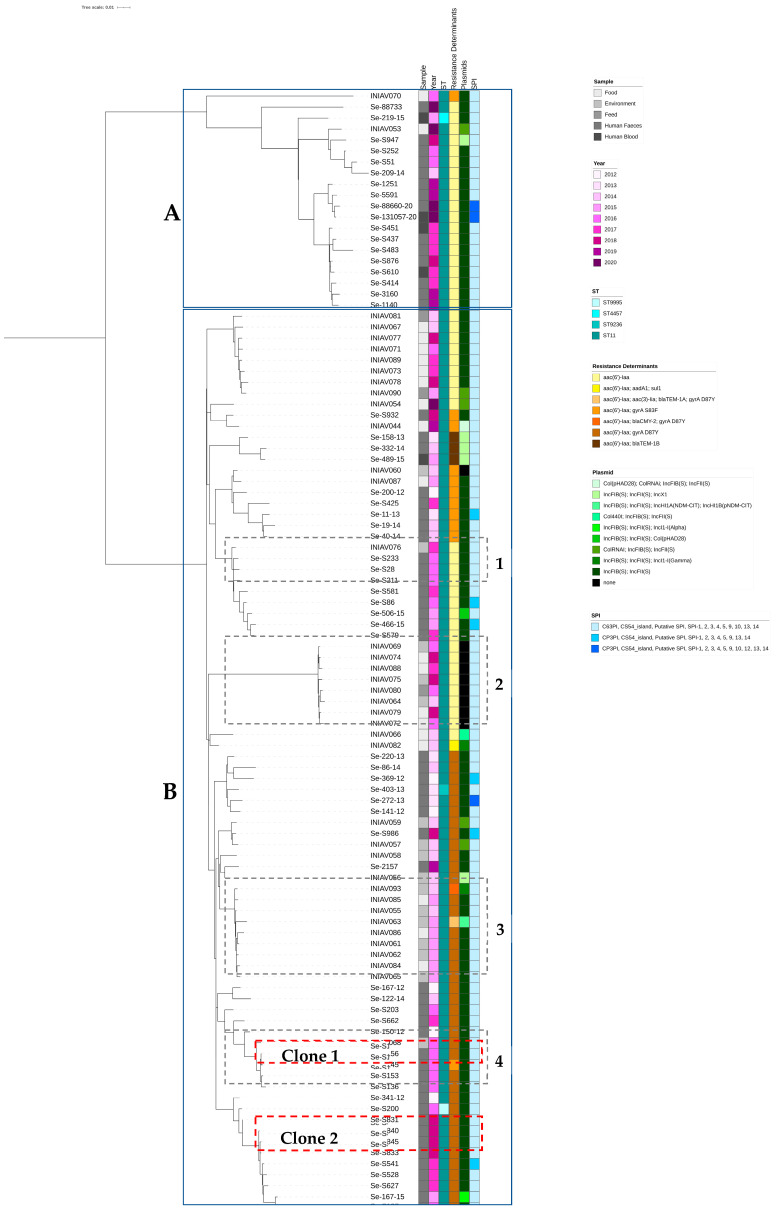
Phylogeny of the *S.* Enteritidis isolated from human and non-human sources with the representation of the genome content of each strain. A phylogenetic analysis based on the maximum likelihood method of the 102 strains from different origins and collection years was performed using *S*. Enteritidis NCTC13349 as the reference genome. The main tree was divided in two major phylogenetic groups: (**A**,**B**). Within group (**B**), four clusters (cluster 1, sharing between 13 and 17 SNPs; cluster 2, 7–17 SNPs; cluster 3, 7–27 SNPSs; and cluster 4, 10–17 SNPs) and two clones (clone 1, sharing 3 SNPs; and clone 2, 4–5 SNPs) were identified.

The genotypic traits of the 102 isolates, including their resistome and mobilome, are summarized in Figure 3. Four different MLST profiles were identified. Most genomes belonged to ST11 (97.1%, 99/102), while three isolates of human origin were novel STs, assigned as ST9236, ST4457, and ST9995 (Figure 3). 

Acquired resistance genes to β-lactams were identified in isolates resistant to ampicillin, namely *bla*_TEM-1A_ and *bla*_TEM-1B_. In addition, the *bla*_CMY-2_ gene was detected in one isolate recovered from an environmental sample resistant to third-generation cephalosporins and cephamycins. All isolates carried *aac(6′)-Iaa*, a cryptic gene encoding resistance to aminoglycosides. Genetic determinants *sul1* and *aaDA1* were identified in one isolate from turkey meat resistant to sulfamethoxazole and streptomycin. Furthermore, chromosomal mutations on *gyrA* (D87Y; S83F; S83Y), responsible for the decreased susceptibility to quinolones, were found in 51% (52/102) of isolates. No plasmid-mediated *mcr* determinants or mutations in the chromosomal genes *pmrD*, *arnE*, *arnC*, *phoP*, *phoQ*, *mgrB*, and *acrB* were found in colistin-resistant isolates. Other chromosomal point mutations on *gyrA*, *gyrB*, *acrB*, and 16S_*rrsD* were identified but were not associated with specific phenotypes. No plasmid-mediated quinolone resistance mechanisms were detected (Figure 3). 

Several plasmid replicons were identified (Figure 3), and IncFIB and IncFII were the most prevalent in 91.2% (93/102) of isolates. Additionally, isolates from human origins harbored plasmids like IncX1 (6.3%; 4/63), IncI1-I(Alpha) (1.6%; 1/63), and Col(pHAD28) (1.6%; 1/63), while isolates from food and environmental sources carried IncX1 (2.6%, 1/39), Col(pHAD28) (2.6%, 1/39), ColRNAi (15.4%, 6/39), IncHI1A(NDM-CIT) (2.6%, 1/39), IncHI1B(pNDM-CIT) (2.6%, 1/39), Col440I (2.6%, 1/39), and IncI1-I(Gamma) (5.1%, 2/39). No plasmid replicons were found in nine isolates (8.8%, 9/102) from food and environmental samples. One IncI1-I(Gamma) plasmid was predicted to carry the *bla*_CMY-2_ and *sul1* genes, and the other carried the *aaD* resistance genes. 

Several SPIs were found in the studied isolates; SPI-1, 2, 3, 4, 5, 9, 13, and 14, C63PI, CS54_island, and Putative SPI were present in all isolates. Most isolates (89.2%; 91/102) presented SPI-1, 2, 3, 4, 5, 9, 10, 13, and 14, C63PI, CS54_island, and Putative SPI (Figure 3). 

**Figure 4:**
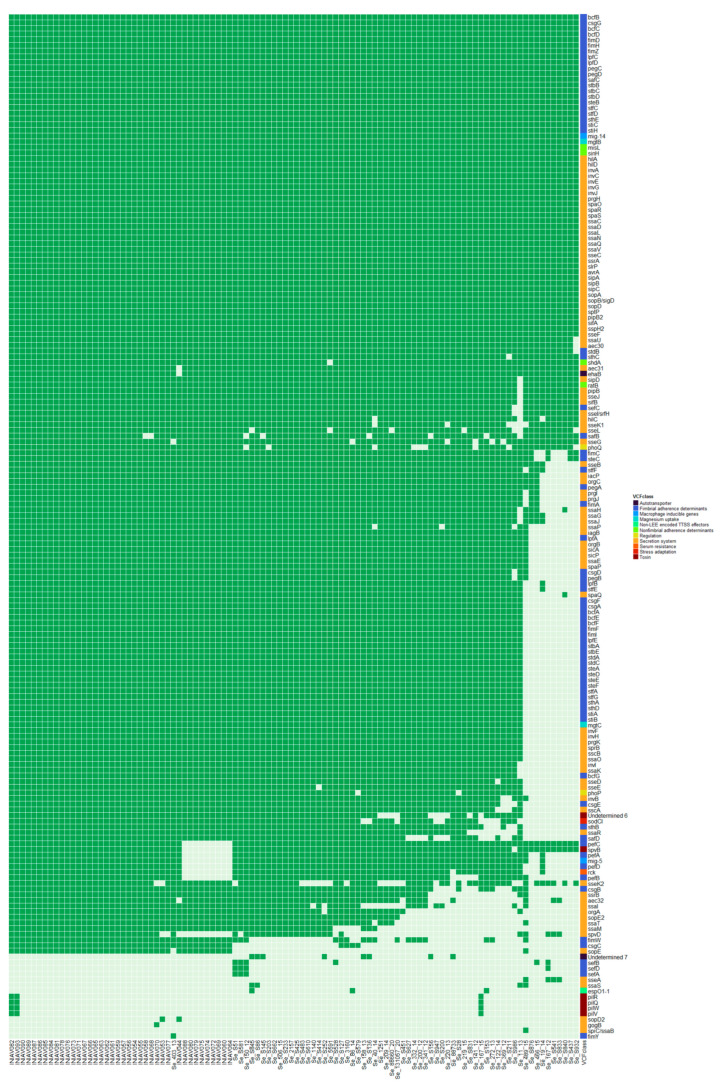
Heatmap representing the presence (dark green) and absence (light green) of genes related to virulence factors (VFs) in strains from human and non-human sources. Genes are ordinated from higher to lower presence according to a presence/absence matrix in at least one strain. Virulence factor classes are represented in colors at the right end.

According to the Virulence Factor Database (VFDB), 170 genes from 11 different virulence factor (VF) classes belonging to nine categories were identified in at least one strain from the set of genomes (Figure 4). Fifty-seven genes associated with five VF classes were found in all genomes. All the isolates from non-human sources presented more VFs than those from human origins, except for isolate Se-403-13. Three genes related to two VF classes, namely *fimY* from the fimbrial adherence determinants class and *gogB* and *spiC/ssaB* from the secretion system class, were only present in one isolate each (Figure 3). The gene encoding exotoxin SpvB was absent in 10 isolates, one from human sources and nine from non-human sources, the same nine isolates that lack plasmid replicons. These nine isolates also lack several virulence genes usually carried by virulence plasmids, such as *pefABCD*, *mig-5*, and *rck*, which are present in most other isolates. The *fimA* gene was present in 92.2%, except for eight isolates from human sources. The genes *spvC* and *stn* were absent in all isolates. 

A phylogenetic tree (rectangular midpoint root representation) was generated based on the SNPs identified between the strains from this study and the reference genome. All strains shared between 3 and 726 SNPs against the reference genome. The genomes were grouped into two major phylogenetic groups (Figure 3); one group was composed of human clinical strains and two strains from food sources (Figure 3A), while the second group was formed by the remaining strains (Figure 3B). Inside phylogenetic group B, it was possible to distinguish two phylogenetic clones (Figure 3, clones 1 and 2, sharing between 3 and 5 SNPs) with only isolates from human sources and two phylogenetic groups (Figure 3, clusters 2 and 3, sharing between 7 and 27 SNPs) only comprising non-human strains, one of which showed no plasmid replicase (cluster 2). Interestingly, two phylogenetic groups with strains from human and non-human sources can be observed. One group had three strains of human origin collected in 2016 and one from an environmental sample collected in 2017 (cluster 1, sharing between 13 and 17 SNPs). The second mixed phylogenetic group, represented in cluster 4, had one strain from an environmental sample and four strains from human feces, all collected in 2016 (sharing between 10 and 17 SNPs). Overall, no relationship was observed regarding sample type, collection date, or genetic content. 

**Figure 5:**
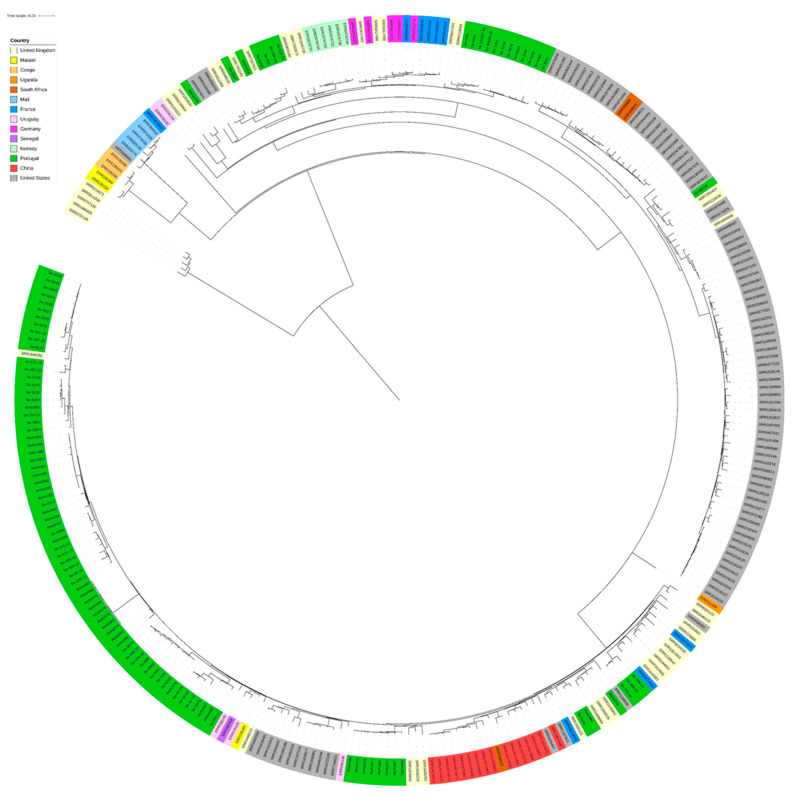
Phylogenetic tree based on the maximum likelihood method, with the representation of the 299 *S.* Enteritidis at a global level showing no genetic relationship according to the geographic region. Different countries are represented with different colors (our strains are colored in dark green).

In a global context, a circular phylogenetic tree (Figure 5) was generated using our 102 genomes and the 197 genomes from a previous study [27], which shared between 1 and 1910 SNPs. Two phylogenetic groups were observed, one comprising five genomes from the UK and the second group with the remaining 294 genomes. In general, the Portuguese isolates grouped, but a few isolates grouped with strains from the UK and US. No genetic relationship was observed according to the geographic region. 

## 4. Discussion

*Salmonella* Enteritidis is responsible for many national and international foodborne outbreaks. It is crucial to understand each country’s population structure and genomic epidemiology [32], as disease severity and antimicrobial resistance profiles vary between countries. In this study, we investigated 102 isolates of *S.* Enteritidis from human and non-human sources regarding genomic characterization and phylogenetic relationships. 

Emerging threats, such as antimicrobial resistance, are among the highest public health priorities within the EU. Overall, antimicrobial resistance in *S.* Enteritidis is lower, compared to other serotypes, namely *S.* Typhimurium, monophasic Typhimurium (1,4,[5],12:i:-), and Infantis [33]. The *S.* Enteritidis population circulating in Portugal was mainly resistant to fluoroquinolones caused by point mutations in DNA gyrase and topoisomerase IV genes; no plasmid-mediated quinolone resistance (PMQR) determinants were detected, although MIC values for ciprofloxacin were low (≤0.5 mg/L). Previous studies on *S.* Enteritidis from poultry in Portugal highlighted the high frequency of resistance to fluoroquinolones, particularly in broilers, breeders, and poultry meat and meat products [34,35,36]. Although the consumption of fluoroquinolones in food-producing animals in most EU countries, including Portugal, has been decreasing for the last 10 years, this class is still widely used in the poultry sector and administered through feed and drinking water for metaphylactic and prophylactic purposes, thus contributing to the selection of resistant isolates [37]. Fluoroquinolones, such as ciprofloxacin, remain one of the most important antimicrobial classes to treat severe human salmonellosis, along with extended-spectrum cephalosporins, such as ceftriaxone [38]. In our study, one strain from a poultry farm environment showed resistance to extended-spectrum cephalosporins and fluoroquinolones. Some prior studies in Portugal also revealed a very low prevalence of resistance to third-generation cephalosporins in *Salmonella enterica* [34,35], with the use of this antibiotic group forbidden in poultry production in the EU [39]. 

Although there is no epidemiological cut-off for colistin established by EUCAST for *Salmonella* Enteritidis, in this study, we used the tentative epidemiological cut-off value (MIC > 2 μg/mL) proposed by Agerso et al. [12]. This cut-off value is also used by EUCAST as a tentative clinical breakpoint and the CLSI as the clinical breakpoint for *S. enterica* used for humans. In this study, a high frequency of colistin resistance in the non-human strains was observed (84.6%). However, this frequency may not represent the real prevalence of colistin-resistant *S.* Enteritidis and can be attributed to the biased selection of bacterial isolates, as one of the selection criteria for sequencing was resistance to colistin. Previous studies in Portugal [40] revealed a prevalence of 23.4% for *S.* Enteritidis. Resistome analysis of colistin-resistant strains did not show any acquired mechanisms of antimicrobial resistance nor mutations in chromosomally encoded mechanisms associated with colistin resistance in Gram-negative bacteria, namely PmrA/PmrB, PhoP/PhoQ, ParR/ParS, ColR/ColS, and CprR/CprS, two-component systems, and alterations in the *mgrB* gene [41]. This serovar belonging to group D, which possesses O:9 somatic antigens, has recently been described as the O-antigen epitope that may govern the levels of colistin susceptibility [42]. Therefore, the epidemiological cut-off value for this antibiotic should be revised for this serovar. 

Other genes of great public health concern identified in several isolates included *bla*_TEM-1A_, *bla*_TEM-1B_, and *bla*_CMY-2_. These genes have also been previously detected in Portugal in food-producing animals, food products, and imported poultry meat (Clemente et al., 2013; 2014; 2015 [34,35,36]) [43]. The *aac(6’)-Iaa* gene was present in the *S.* Enteritidis population analyzed and did not correlate with resistance as expected, since aminoglycoside acetyltransferases genes are often weakly expressed or not expressed [44]. 

The identification of several plasmid replicons, including IncFIB, IncFII, IncX1, IncI1-I(Alpha), and IncI1-I(Gamma), among others that have been associated with harboring antibiotic resistance genes [45,46], suggests that the Portuguese *S.* Enteritidis populations could harbor and probably disseminate antibiotic resistance genes via plasmid-mediated gene transfer. 

The most frequent MLST profile of *S.* Enteritidis found circulating in Portugal was ST11 (97%), and the three novel STs (ST9236, ST4457, and ST9995) were associated with human strains. *Salmonella* Enteritidis ST11 seems to be one of the most prevalent sequence types [32] and has been detected in various reservoirs, including humans, poultry, and food sources, with a wide geographic distribution spanning Europe [1,47], Asia [48], Africa [47,49], and South America [50]. 

In this study, several *Salmonella* pathogenic islands (SPI) were present in the *S.* Enteritidis genomes, namely SPI-1, 2, 3, 4, 5, 9, 10, 12, 13, and 14, C63PI, CS54_island, Putative SPI, and 170 virulence genes belonging to nine virulence categories were identified. Pathogenic islands are large genomic insertions only present in the genome of pathogenic bacteria, encoding multiple virulence factors crucial for survival in the host cells. The virulence genes are responsible for the bacteria’s invasion, survival, and spread and are acquired by horizontal gene transfer, usually related to mobile genetic elements [51,52]. Some SPIs are highly conserved regions between the different *Salmonella* serotypes, namely SPI-1, ubiquitous among all subspecies and associated with the invasion of the host cell; SPI-2, just present in *S.* enterica and associated with the survival within the macrophage; and SPI4, associated with the secretion system. SPI-3 and SPI-5 can be variable regions within *Salmonella* spp. and are associated with survival in the intra-phagosomal habitat and systemic infection, respectively. SPI9 and SPI10 are involved in the adhesion process. The roles of the regions of SPI-12, 13, and 14, CS54, and the pathogenicity island of centisome 63 (C63PI) are not well known but also associated with survival and adaptation within host cells [51,52]. 

The presence of the *spv* operon (namely *spvB*, *spvC*, and *spvR*) and the genes *inv*A, *hil*A, *fim*A, and *stn* are usually associated with severe infections in non-typhoidal *Salmonella* [53,54,55]. In the current study, 100% of strains carried the genes *invA* and *hilA*, 92.2% harbored the *fimA* gene, and 90.2% carried the *spvB* gene. Our findings demonstrate the potential pathogenicity of the *S.* Enteritidis strains isolated from human and non-human sources, which is in line with what has already been described by other authors [56,57,58]. 

Overall, the phylogenetic tree revealed that strains of human and non-human origins from Portugal are genetically heterogeneous regarding sample type, collection date, or genetic content. Even so, strains were grouped into two phylogenetic groups, both with strains from different sources, and several clusters were identified. The two clusters comprising only non-human strains (clusters 2 and 3) corresponded to different types of samples (boot swabs, food, feed, meat, etc.), with different collection dates, different origins, and different species of poultry. Due to a lack of epidemiological data, no correlation between strains from cluster 1 can be established. The strains from human sources of cluster 4 were part of an outbreak identified in July 2016 potentially associated with artisanal ice cream. SNP analysis revealed that two of these strains were clones (clone 1). Interestingly, a sample from a boot swab collected in a laying hen facility in May 2016 was clustered with these strains. No information about the source of the ice cream ingredients was available, making it difficult to establish any relation between these strains. Finally, another clone was identified, clone 2, comprising three strains sharing four and five SNPs, all isolated from children aged 5–8 years from the same period, although other epidemiological data are missing. 

*Salmonella* Enteritidis populations have remarkable genetic diversity, as revealed by phylogenetic analysis of single-nucleotide polymorphisms (SNPs) [47]. When comparing the strains from this study with *S.* Enteritidis strains from other countries published in a previous study [27], most strains from Portugal (80.4%, 82/102) were included in the well-established global/cosmopolitan clade [27,47]. Similar to what was described for other geographical regions, it is even possible that a clone has persisted and evolved in Portugal, since most strains (75.6%, 68/82) form a subclade within the global/cosmopolitan clade [27]. Also of note is that MDR profiles and the presence of *bla* genes were exclusive to the strains in this clade. The remaining 20 strains from this study were distributed through the Outlier/Atlantic clade, grouping with isolates from the US or UK.

## 5. Conclusions

In this study, a whole genome sequencing analysis was performed using 102 *S.* Enteritidis strains from human and non-human sources to characterize the genetic diversity of *S.* Enteritidis circulating in Portugal from a One Health perspective. 

Our findings revealed that the *S. enterica* Enteritidis population studied was mainly resistant to fluoroquinolones, demonstrating that antimicrobial resistance in *Salmonella* may also be a concern in Portugal, as observed in other countries. 

ST11 was the most frequent MLST profile circulating in Portugal, with three novel STs from human strains (ST9236, ST4457, and ST9995) being described. Our findings demonstrate the potential pathogenicity of the *S.* Enteritidis strains isolated from human and non-human sources, due to the number and type of virulence genes identified in the genomes. 

The phylogenetic analysis revealed that these strains of human and non-human origins from Portugal are genetically heterogeneous regarding sample type, collection date, or genetic content; in a global context, the Portuguese strains grouped, but a few grouped with strains from the UK and US. With this study, we are increasing the available information and genomic data of the *S.* Enteritidis population circulating in Portugal for the scientific community, contributing to a better understanding of the genomic characterization of strains in Portugal and at a global level.

## Data Availability

The data supporting this study’s findings are available within the article.
